# Grief in Response to Uncertainty Distress Among Veterinary Students During the Early Stages of the COVID-19 Pandemic

**DOI:** 10.3389/fvets.2021.662198

**Published:** 2021-07-08

**Authors:** Kimberly Carney, R. Randall Thompson

**Affiliations:** College of Veterinary Medicine, Lincoln Memorial University, Harrogate, TN, United States

**Keywords:** COVID-19, well-being, stress, veterinary student, uncertainty

## Abstract

The abrupt and life-altering shifts brought on by the COVID-19 pandemic have stimulated research in fields ranging from social sciences to virology. This study explored perceptions and experiences of COVID-19's impact on students at Lincoln Memorial University–College of Veterinary Medicine (LMU-CVM) and considered how to respond to these. Semistructured interviews were conducted with 20 students from LMU-CVM. Thematic analysis elucidated five subthemes that were combined into two main themes based on Bertuccio's framework of grief in response to uncertainty distress. Uncertainty and disruption of routine were subthemes of ambiguous loss, while lost opportunities, milestones missed, and risk concern came under anticipatory grief. There was overlap and fluidity within these themes, with frustration, stress, and unexpected benefits pervading all categories. Differences were noted between classes, with clinical students expressing concern over graduation and lack of preparedness, and preclinical students with online assessment, lost opportunities for clinical experiences, and the loss of social connections. These results point to mitigation strategies for the adverse effects of COVID-19-related stressors specific to this population that encompass academic, physical, and mental well-being concerns. Clear communication, assurance of quality education, flexibility for meeting family needs, financial assistance, and mental health support are the areas evident from the interviews where successful responses might be targeted. This study also highlights areas for future research, including follow-up interviews, given the prolonged timeline of COVID-19, surveys of beliefs and practices across a larger university population, and exploration of the long-term impact on academic and practice success of the affected cohorts.

## Introduction

The declaration by the World Health Organization of SARS-Cov-2 (COVID-19) as a pandemic in March 2020 brought significant challenges for people everywhere. Public health policies to slow the transmission of the disease caused rapid changes in how populations interacted and conducted daily activities. Travel restrictions, social distancing, and closure of many businesses created an upheaval of habits and lifestyle, causing people to respond to their most basic needs of physical safety and security, which gave rise to panic buying of groceries, personal protective equipment, and cleaning supplies (1, 2). These restrictions included a wide variety of services, and within a few weeks of the declaration, schools and educational institutions worldwide closed or moved to online instruction, impacting over 80% of the world's student population (3). The isolation, uncertainty, and rapid adaptations necessitated by the COVID-19 pandemic have led to significantly higher levels of stress, anxiety, panic, and posttraumatic stress disorder being reported worldwide (4, 5).

The mental well-being of all people has been impacted by COVID-19, but Xiong et al. reported in a systematic review that females, students, and people under 40 years old reported more stress as well as depressive and anxiety symptoms than other groups during COVID-19 (5). This highly affected demographic is represented in the veterinary student population of North America, which is more than 80% female and has an average age under 25 (6). Prior to the COVID-19 pandemic, it had been recognized that veterinary students faced higher levels of stress, anxiety, and depression than the general public or undergraduate students (7, 8). Among graduate and professional students during the pandemic, Chirikov et al. reported a prevalence of major depressive disorder two times that of 2019 and generalized anxiety disorder 1.5 times higher than in 2019, implying that COVID-19 has worsened these conditions (9). This prevalence was even higher in those students who reported that they “did not adapt well to remote instruction (9).” It is important to note that these were self-reported measures of depression and generalized anxiety, not medical diagnoses.

Rather than large-scale diagnoses of mental health disorders, Bertuccio proposes that as a response to the pandemic, these potentially subsyndromal expressions of depression and anxiety could be characterized as grief experienced in response to uncertainty distress (4). Uncertainty distress is defined as “the subjective negative emotions experienced in response to the as yet unknown aspects of a given situation” and can be used to normalize responses to abnormal circumstances (4, 10). This lens of grief contextualized by uncertainty distress “may be useful to study reactions to the pandemic (4).” In a population like veterinary or medical students who have reported stigma around mental health diagnoses, grief and uncertainty distress may be less stigmatizing than a diagnosis of depression or anxiety, enabling earlier recognition and treatment (11–13). This is not meant to diminish the needs of those with diagnosed depression or anxiety disorders but to recognize the acute, temporal, and situational effects of the COVID-19 pandemic.

The framework of grief in response to uncertainty distress can be divided into three main components: ambiguous loss, anticipatory grief, and complicated grief (4, 10). Ambiguous loss is defined by uncertainty and causes unresolved grief. This has been indirectly expressed among medical students during COVID-19 in editorials clamoring for transparency, communication, and continuity in the quality of their education (14). It is the result of not knowing when or if normalcy will resume. Anticipatory grief is that of a dreaded future loss or grieving what is expected to come (4). As an example, during the pandemic, human medical students have voiced concerns about the long-term impact on careers if they are unable to practice live patient interactions, about graduating on time, and about residency matches and their preparedness for practice (15). Complicated grief has severe and dysfunctional symptoms of grief that appear similar to anxiety or depression but is in reaction to loss. Symptoms of this include longing for what was lost, loneliness, shock, anger, and mistrust that often arise from circumstances outside of the individual's control, like the inability to hold funeral services or attend support groups during social distancing (4). As the life changes associated with the pandemic continue over many months, complicated grief could arise.

It is hypothesized that COVID-19 has caused further detriment to the mental well-being among veterinary students. The aim of this study was to use individual interviews to investigate the impacts of the COVID-19 pandemic on the mental well-being of veterinary students at LMU-CVM in their personal, academic, and professional lives. A secondary aim was to identify areas of distress the university administration and faculty could mitigate to improve student well-being.

## Materials and Methods

### Setting, Study Design, and Participants

Lincoln Memorial University–College of Veterinary Medicine (LMU-CVM) has an enrollment of over 400 students. The curriculum is heavy in hands-on laboratory experiences, with students in skills laboratories multiple days per week. As COVID-19 burgeoned into a pandemic, the recommendations for social distancing necessitated dismissing students from in-person classes with a switch to online classes. The timing of the dismissal was <1 week before the scheduled spring break of the college, and with the uncertainty of the situation, the initial communications indicated a possible return to class after spring break. As university policy was made and adapted to ongoing developments, students faced a few weeks of extreme uncertainty until the remainder of the semester was changed to online only.

Clinical (fourth) year students had begun a new rotation location 3 days prior to the decision to stop in-person clinical experiences. LMU-CVM employs a hybrid distributive model for clinical placements, which means there is no referral teaching hospital as is common in most US veterinary schools. In this model, fourth-year students change geographic locations monthly, and their clinical year consists of supervised clinical rotations with approved primary care and specialty referral partners predominantly in the US with a few international options. COVID-19 required these clinical-year students to rapidly change plans, including securing alternative housing to complete their remaining rotations online. Preclinical (years 1–3) students varied in their decisions to remain in the area or return to their permanent residences. At the time of the study, participants had spent roughly 4 weeks in the new online system.

The study was approved by LMU's Institutional Review Board (903V.1). Recruitment occurred via mass email to all enrolled students at LMU-CVM via their university-assigned email address. Responses were collected for 48 h, then five participants were selected at random from each of the four class years for a total of 20 respondents (18 female, 2 male; Table 1). This ratio of female to male is slightly higher than that of LMU-CVM, which averages 20% male. An initial sample size of 20 was predicted to achieve saturation of response as commonly reported in the analysis of qualitative literature (16). This was confirmed during initial coding of the first set of interviews; no new codes emerged from the final six interviews. A subsequent round of interviews was not conducted (17). All participants gave informed consent; confidentiality was ensured for participants through use of alphanumeric coding.

**Table 1:** Patient demographics.

**Group**	**Female**	**Male**
1st-year students	3	1
2nd-year students	6	0
3rd-year students	4	1
4th-year students	5	0

### Interviews

Semistructured interviews were conducted from April 17 to 30, 2020, 1 month after declaration of the pandemic. The authors worked together to create the interview questions and process. The interview guide is available in the Supplementary Materials; the flexibility to change the sequence and phrasing of questions was retained. Participants' interpretation, understanding, and meaning were clarified during the interviews through prompts such as “Tell me more about that” or “What did you mean when you said…?” Each author conducted one-on-one interviews with roughly half of the respondents; these were conducted via Zoom (Zoom Video Communications, San Jose, CA, USA) and lasted from 25 to 48 min. These were securely recorded and digitally transcribed via Zoom and maintained on the primary author's Zoom account until downloaded to the authors' password-protected computers. The authors reviewed transcriptions to ensure accuracy and confidentiality. Transcriptions were deleted from the Zoom cloud upon downloading to the physical computers. Participants were able to withdraw from the study, without giving any explanation; none chose to do so.

### Theory and Analysis

A thematic analysis (18) of the recorded and transcribed interviews was conducted by the first author, and validity was ensured through a continuous process of reflection and discussion with co-author to establish consensus on the final coding and interpretation. Codes were identified from the data in an inductive approach, not informed by an *a priori* framework. Semantically identified initial codes were analyzed for and organized into themes (underlying ideas, assumptions, and conceptualizations) and subthemes. The themes that were identified were then mapped onto Bertuccio's framework of grief as a response to uncertainty distress as described in the results (4). See Table 2 for a sample of codes, subthemes, final themes, and exemplar quotations.

**Table 2:** Themes and coding exemplars.

**Final Theme**	**Subtheme**	**Initial code**	**Exemplar quote**
Ambiguous loss	Uncertainty	Communication	I think the uncertainty of what's going to happen is probably the biggest stressor, not knowing everything going to get back to normal. S2
		Academic stress	But since COVID started, just the anxiety of worrying about everything has made it extremely hard to focus. My motivation to study has, like, bottomed out. S18
	Disruption of routine	Changes in schedule	It's been difficult on my like anxiety and depression, not being able to like basically being thrown out of routine. S13
		Social interactions	I'm usually an introvert, so I didn't think it would bother me this much, but being cooped up for over a month now I'm like ready to see my friends and go and do things. S8
		Free time	But it's nice to kind of have my own schedule. I can go to the barn in the middle of the day and then watch lectures in the evening. S14
		Work/home separation	They put the rest of the semester online. When that happened, I was like, okay, I can't stay here at home because there's too many distractions. S3
Anticipatory grief	Lost opportunities	Canceled labs/rotations	I feel more like I'm watching an educational video versus being a part of it in clinical skills. S20
		Lost summer experience	One thing that's been getting to me a lot, is that I had a job pretty much lined up for the summer and they are no longer taking students. So that's gotten me very stressed lately. S1
	Milestones missed	No commencement	The class is really upset about, you know, not having like a climactic end to our clinical year. Everybody's really disappointed about graduation. S7
		Missed family events	My friend just had a baby. It's been 3 months and I haven't been able to meet her. S11
		Lack of closure	Not being able to say goodbye to my friends is difficult and it just kind of puts like a, I don't know, it's just like a depression about everything. S11
	Risk concern	Professional impact	I wasn't able to retain information as well. S8
		Social distancing/mandates	It's kind of like being in kindergarten and another student does something wrong and it kills your recess time. You're like, dude, you can't just follow the rules for 5 min? S2
		Financial/economic concerns	Eventually we want to buy a house and not having to have that [unemployment due to COVID] on our record would be good. S4
		Disease risk–self	You never know, like a very healthy person could contract it and it wipe them out entirely. S11
		Disease risk–others	I'm concerned I'm going to bring something back and it's going to do something to my family, not so much concerned for my own well-being. S17

To ensure continuity in the interview analysis, codes and themes were again discussed with both authors and reviewed and approved by the participants. The authors/interviewers were both clinical faculty with whom all students interact frequently and who strongly value student well-being.

## Results

Using Bertuccio's framework of grief as a response to uncertainty distress, two final themes were defined: ambiguous loss and anticipatory loss. Since the interviews were conducted within the first month of the pandemic, the prolonged impact of complicated grief is anticipated but not yet defined. Within these final themes, the subthemes included the following: (1) uncertainty, (2) disruption of routine, (3) lost opportunities, (4) lost milestones, and (5) risk concern; these are shown Table 2. Every theme was noted in at least one individual in each group, but the relative importance and frequency shifted between clinical and preclinical students.

There was overlap and fluidity within these overarching themes, and the frustrations of increased use of technology infiltrated conversations. Frustration, stress, and surprising benefits were commonly expressed throughout the interviews, illustrating the complex interplay of emotions and life challenges during such a time of uncertainty. This complexity was expressed as a shared sense of community and acknowledgment of the universality of the situation.

### Ambiguous Loss

#### Ambiguous Loss: Uncertainty

Underlying many of the strong emotions around the COVID-19 pandemic was uncertainty. This uncertainty can lead to unresolved (and often unrecognized) grief as the anxiety and psychological losses caused are ambiguous. Anxiety and the perception of grief stem from a person's intolerance of uncertainty more than the uncertainty itself (10).

Uncertainty about the disease itself and what changes to expect pervaded the results. The few weeks before the interviews were especially trying as communication changed rapidly both about the disease and the university's response to it. The lack of clear understanding made planning anything difficult. Student 2 expressed, “I think the uncertainty of what's going to happen is probably the biggest stressor, not knowing when everything is going to get back to normal.”

Students finishing their third year (5/5) seemed most impacted by uncertainty as their first few clinical rotations were going to be online and their carefully planned clinical-year schedules were suddenly upended. The uncertainty about their clinical-year scheduling and the shifts in schedule brought emotional and financial stress. Housing and travel plans had to be changed rapidly, often with lost deposits, as clinical sites were unable to accept students for at least the first three rotations, and the status of the remainder of their clinical year was uncertain. Clinical-year students expressed uncertainty about completing online job interviews, with student 6 commenting, “I didn't want to accept a job somewhere I've never been and seen,” referring to a veterinary practice that did not conduct in-person interviews during COVID-19. There was even concern about in-person interviews not showing the true nature of a practice with the limited client interactions observed due to social distancing. Those who already had secured placements were uncertain about their employment status, start dates, and if the practices would still be open and functioning.

#### Ambiguous Loss: Disruption of Routine

Cancellation of in-person classes, coupled with safer-at-home restrictions forced students into different routines for both personal and academic life. The disruption of routine threaded through many of the conversations and other themes as a vaguely or acutely felt loss. Students were asked about stressors from before COVID to during COVID. These shifted dramatically for many respondents but were mostly related to disruption of their routines. School-related stress was the most mentioned pre-COVID stress, but during the pandemic, daily stress shifted to COVID-related challenges, as student 16 divulged, “doing normal things, like grocery shopping, has become stressful, which is just bizarre.”

The change in going to work or school and having a clear time for tasks led to shifts in exercise routines, study habits, and overall well-being. “It's been difficult on my anxiety and depression…being thrown out of routine, and so it's something I've really struggled with,” admitted student 1.

Moving from a very hands-on atmosphere to online learning was seen as a significant disruption. Some lectures had already been online, but having all assignments, including group work, online was a challenge. Differences in technology adaptation between professors, the use of multiple platforms, and different time zones were cited as major issues:

It's hard because they're not in the same time zone, so group projects have definitely been one of my major things that I hate about online. I don't want to do it again (S19).

Engagement in asynchronous classes challenged some, as student 20 mentioned, “I feel more like I'm watching an educational video vs. being a part of it.” The blending of academic life with personal or home life was abrupt and frequently mentioned (13/20). Participants fell on both ends of the spectrum with regard to time management—some found challenge in staying motivated to study or work at all, while others had a hard time partitioning their time and were studying even longer hours than before. Home was no longer a sanctuary away from work or study. Students commonly (16/20) mentioned that while at home they struggled because they were not in “a committed learning environment,” as student 18 labeled it. While contact with and the support of family or roommates is usually positive, being thrust into nearly constant contact with just a few people with different schedules and responsibilities was trying. A lack of personal space, makeshift offices, interpersonal conflict, and intermittent internet were frequently (14/16) cited challenges. From sharing limited laundry facilities to finding a private place to meet virtually with counselors, stay-at-home arrangements raised uniquely challenging circumstances for many. Student 13 complained, “Family was rough trying to get schoolwork done.” Households often had more people than usual, making quiet spaces for studying and online exams challenging. Upon returning to her family's home, student 3 discovered, “My family was in the middle of remodeling the house. So, my bedroom wasn't even usable. It was a closet.”

These challenges were exacerbated by the change to online exams. Preclinical students (9/15) reported anxiety over the online exam proctoring system. The system caused students to worry about every noise their families made, about pets that walked through the room, even that their appearance was different and would flag them: “I made sure I showered and dressed up [for a practice exam], and then I realized that is not how I look when I take exams” (S17). Inconsistent internet availability added another layer of stress to online exams as students wondered, “Are they even going to get my exam?” (S19).

The disruption of routines extended beyond physical living space to disruptions of social interactions. The sudden and prolonged end of face-to-face interactions has had a profound effect on relationships and support systems. Even the self-proclaimed introverts (9/20) report missing social events. Student 3 remarked:

It's definitely taking a toll, which is interesting because I've always kind of thought that was more of an introverted person but realized now through this that I rely more on that personal interaction and connection with people than I realized.

Communication shifted online through many platforms, but students missed the freedom to go out to eat or “grab a drink” with friends. Online happy hours, interactive game apps, and online meeting platforms enabled some continuity of relationships, with student 10 remarking, “We always went to Thursday night trivia at a bar, so we do Zoom trivia now.” Beyond peer groups, most participants (16/20) mentioned missing informal faculty–staff–student interactions.

On the positive side, those with family at home (significant others, children, and pets) learned to enjoy the slower pace and limited contact. Student 8 conveyed, “We…learned it's okay to stay home on weekends and not have plans and just hang out with the dogs and just us [family].” Others (4/20) that had been in long distance relationships throughout school were able to spend time together.

### Anticipatory Grief

#### Anticipatory Grief: Lost Opportunities

The perception of lost opportunity was evident in the academic and professional realm. Nearly every preclinical student (11/15) remarked about missing the clinical skills labs that were hands-on and very interactive with professors. They expressed concern over the long-term impact of reduced experience in labs and classrooms leading to reduced readiness to practice and being insufficiently prepared for national licensing exams.

Summer internships, leadership experiences, and overseas programs were canceled, leaving students with gaping holes in their summer plans and expectations. While they mourned these lost opportunities, most students optimistically followed it with a comment like student 15's, “It could be a lot worse. There are people that don't have jobs, and there's people that are suffering.”

The clinical-year students appreciated the opportunity to graduate on time with the shift to online rotations but felt they were missing valuable experience. While all clinical year had two complete rotations shifted online or canceled, they still felt prepared for practice: “I feel prepared to start my career,” said student 6, and student 7 agreed, “I don't think it'll impact my ability to function as a DVM.”

#### Anticipatory Grief: Milestones Missed—Grandparents, Babies, Weddings, Graduations, Funerals

Social distancing has promulgated emotional challenges in the loss of in-person family interactions. More than half of respondents (12/20) commented about not being able to visit extended family. Student 5 remarked,

I can't go see my grandmother. I go buy her groceries every Thursday, and then I bring them home to her, but like, you know, she opens a glass door and I hand them to her. I haven't hugged her, and I just want to squeeze her. God, I can't stand it.

Those with immunocompromised family have chosen to isolate in other locations to protect them but have really felt the loss of freedom to visit, as student 18 noted, “I think that's been the hardest part [upon dropping off pets with family, wearing mask and gloves] I [air] hugged my dad through the glass French doors.”

While online versions have attempted to fill the gaps, respondents felt loss at missing graduations, homecomings, weddings, birthdays, and first words of nieces and nephews. Student 9 lamented,

I was supposed to go for spring break, we had planned this huge family reunion and my brother was on leave from the military and my mom would be out of the hospital, but, like that got canceled.

One of the most impactful milestones missed was an in-person commencement. Students, especially fourth-year students (5/5), were happy to complete their degrees without delay but mourned not being together to celebrate and bring closure to this stage of life. This grief may have been compounded by the fact that the clinical-year model prevents them from seeing most of their classmates, faculty, and staff during their clinical year. They were not on different rotations in a teaching hospital where they might at least pass in the hallway or meet over lunch; they were predominantly on rotations in different states. Student 7 summed up the sentiment saying, “The class is really upset about not having a climactic end to our clinical year—everybody's really disappointed about graduation.”

#### Anticipatory Grief: Risk Concern

Anticipatory grief was noted in the discussion of students' concerns about the risks associated with COVID-19. None of the students disclosed having had the virus yet, but the anticipation of disease and its longer-term complications impacted them nonetheless. Students grieved the possibility of other friends and family suffering from the disease as well as the ancillary risks to their own futures.

Risk concern of disease transmission directly influenced the motivation for following stay-at-home orders and social distancing recommendations. This included concern for contracting COVID-19 personally and the risk of spreading it to others.

All (20/20) participants reported following safer-at-home and personal protective equipment recommendations strictly, though only two stated they were concerned for their own health. When asked about their motivation for following the recommendations, the most common answer was concern for others, as student 14 remarked, “It's just more who I could spread it to more than worrying about myself necessarily.”

Though the majority (18/20) were not concerned about their personal disease risk, a few were concerned about the larger risks of the pandemic beyond illness. Students considered the impact on both personal finances and the global economy. Student 4 worried, “Eventually we want to buy a house and not having to have that [unemployment due to COVID] on our record would be good.”

### Hidden Benefits

Heightened stress, anxiety, and worry about the future predominated conversations, but there were some hidden benefits brought to light through this time of enforced slowdown. As health professionals, it may have been frustrating to see the public ignoring recommendations, but the “One Health nerd(s)”(S13) and scientifically curious minds of participants were fascinated by watching the science unfold and bring to the forefront the concepts they had been taught.

Adaptations due to the pandemic provided many teachable moments to model resilience, professionalism, quality, and safety while moving bioethical issues from theory to practice (19). For example, students were able to see challenging decisions made to prioritize personal protective equipment for human healthcare over elective animal procedures.

A sense of community or at least shared sorrow pervaded the group. When students commented on long-term impact on their knowledge or skills, it was often followed with a statement of, “it's not just us, it is students everywhere” (S4). This optimism encouraged people to think more creatively about their time.

The flexibility of scheduling allowed students to use daylight hours to help on family farms, spend more time with their horses, and enjoy more outdoor activities, then spend evenings catching up on schoolwork. The perception of increased free time was used in a multitude of ways. Binge-watching television, exercising, gardening, and learning new skills were common activities.

## Discussion

The overarching idea of grieving perceived losses permeated the responses, thus leading us to use the framework of grief as a response to uncertainty distress as described. Instead of pathologizing responses to the pandemic as new diagnoses of anxiety or depression, the model of grief in uncertainty distress proves helpful in studying reactions using “contextualized grief and trauma lenses.” (4). Though most participants did not explicitly label their experience as grief, it was easily recognized in the final themes of ambiguous loss and anticipatory grief. The potentially unrecognized mental health challenges that result may present as anxiety, guilt, or depression within the typical grieving process model and serve to highlight the importance of providing and normalizing mental healthcare within veterinary colleges. The third component of Bertuccio's framework, complicated grief, could not be assessed in this early cross-sectional study but is anticipated to gain importance as the pandemic and its effects are prolonged.

We suspect these experiences are not unique to the LMU-CVM based on recent publications on COVID-19 impacts across populations (1, 5, 14, 20–22). These early reports on the impact of the pandemic, through a mixture of popular and scientific press, shed light on universal challenges experienced by some populations (see note about references in **Limitations** section). Gallagher et al. reported the “loss of routines and traditions, expertise, educational opportunities, and social connection” among medical students (19). A survey by the nonprofit, Active Minds, reported that university students experienced higher levels of anxiety or stress (91%), disappointment or sadness (80%), and relocation (56%) due to COVID-19 than in previous years. They further related that 76% had trouble maintaining a routine, 63% found it challenging to stay connected to others, and 85% said that focusing on school and work despite distractions has been the biggest challenge of stay-at-home orders (23). These results are similar to the experiences shared by our students. These factors have a significant impact on mental well-being, especially for those with preexisting mental health conditions. The loss of routine, connectedness, and limited access to supports like counselors can exacerbate conditions, as student 1 expressed. LMU-CVM already offered telehealth counseling services for traveling clinical-year students but expanded this coverage to all students to meet their needs during the pandemic.

LMU-CVM clinical-year students (and rising clinical students) faced quite different challenges than those of many veterinary schools with traditional teaching hospitals. The added stress of housing cancellations and rearranging of travel plans created more uncertainty as compared to other CVMs in which students remain in the same location all 4 years. After the initial 3 months of severe restrictions, the LMU-CVM model proved advantageous as students could be spread out across many practices in different areas that were open sooner than most traditional teaching hospitals.

One of the important lost opportunities that can lead to complicated grief is marking milestones with family and friends. The loss of these milestones was noted throughout the interviews pertaining to births, funerals, family reunions, and, especially, commencement. These celebrations are an integral part of society that have been missed during the pandemic. In her article, “The Importance of Celebrating Milestones Together,” (24) psychologist Dr. Marie Hartwell-Walker comments:

Ritual celebrations are important because they provide structure and predictability in an unpredictable world, help people make important transitions, foster and affirm connection, provide models, create memories, and preserve culture.

The COVID-19 pandemic has certainly created an “unpredictable world” and changed our ability to celebrate those rituals. LMU-CVM, like many institutions, held virtual commencement and student engagement opportunities, but these could not fully replace the emotional connection with in-person events and the major life transitions they mark. The continued absence of milestones together is predicted to make complicated grief more prevalent as the pandemic and its aftermath continue (4).

### Response

Supporting students as the effects of COVID-19 continue should involve a holistic approach to encompass academic, physical, and mental well-being concerns. The concerns expressed by the students interviewed were mirrored by students in other healthcare professional programs. Medical students at the University of Washington reported student apprehension about lack of skills, on-time graduation, financial aid, and practical concerns like child care (19). Clear communication, assurance of quality education, flexibility for family needs, financial assistance, and mental healthcare are obvious channels of support for students through the COVID-19 recovery process. Important considerations to facilitate these include increased technological and academic support, increased availability of mental health resources, empathy, opportunities for social connection, and long-term planning (23).

Clear communication within the university was cited by Makenzie Peterson, AVMA Well-being Director, as a critical component to the well-being of all members, so that there was a “clear rally point” for people to understand (25). Creating a sense of stability and continuity in the long-term future of an academic program reassures students that their professional plans can be completed. Transparent decision making, with some explanation of the rationale, can help students understand and accept the importance of administrative decisions. To that end, LMU-CVM increased the frequency of the mandatory “Dean's Class” from monthly to weekly during the pandemic to ensure timely communication. Student needs regarding educational quality and mental health were monitored via QR code-linked surveys after each lab and weekly Qualtrics surveys of physical and mental health during the Dean's Class. In addition to the Dean's Class to address university policy and student concerns, the One Health faculty held weekly updates on the scientific concerns and developments of COVID-19.

Recognizing that the initial shift to online learning was an emergency measure, future semesters should benefit from thoughtful planning and adopting best practices for online delivery. Dr. Elizabeth Strand reminded faculty to have “grace with themselves and empathy for students as they convert programs to online delivery, but cautions…to maintain quality (25).” Comprehensive planning of delivery methods, adapting potential long-term social distancing protocols, and planning for the need for social interaction of faculty, staff, and students will be the challenge for schools as the pandemic continues and after.

Supporting immediate physical needs like financial aid, housing, and education should be combined with supports for mental well-being. Beyond faculty modeling adaptive behaviors, institutions should seek to normalize mental well-being challenges and treatment. Especially in treatment of uncertainty distress, seeking to normalize concerns as expected responses to the current uncertain circumstances can destigmatize mental healthcare (10). Understanding the grief response as an expected response to the situation can reduce the barrier for students to seek mental healthcare. With many students not physically on-campus, accessing counselors may be challenging. Telehealth counseling provides a safe, confidential, and accessible alternative that universities can offer their students.

Encouraging development of resilience, recognizing the potential for posttraumatic stress, and providing appropriate treatment are necessary for recovery from the pandemic. In many cases, the act of introspection during the interview led participants to recognize positive aspects alongside the initial negative responses. Mindfulness exercises and reflective journaling could be incorporated to develop student coping behaviors (26).

Understanding the lived experience of different populations through the COVID-19 pandemic will enable educators to better support students during the continued changes and throughout the recovery process. The shake-up of systems and values can serve as a launching point for innovation and lasting improvements in policy, process, and community. It may also position all of us to be better prepared to deal with the unforeseen challenges of the future.

## Limitations

There can be little doubt that the timing of the interviews influenced the responses. These were conducted 4–6 weeks after the pandemic was declared and a few weeks before the end of the semester. Most students seemed to have forgotten the initial frustration of rapidly changing communication and were deep in preparation for final examinations and end-of-semester assignments. Clinical-year students were mostly finished with requirements for graduation and had little responsibility.

There could be selection bias toward students who had ready internet access and checked email frequently. Self-selection by students interested in the research topic, open to talking about mental health, or knew the researchers could bias the sample selection.

The body of literature related to COVID-19 pandemic impacts on students was limited by the novelty of the topic. Some of the references cited were from popular press and editorial comments.

Many of the experiences of our students were mirrored in reports from other health professional students; our findings may be generalized to other similar populations with caution.

## Further Research

As the pandemic continues, additional research is necessary to reassess the participants' responses and note changes to better inform the college of the emerging challenges of students. The current questionnaire could be adapted to better understand the effect of the continuation of COVID-19 responses on this population.

Delving more into the mental health implications and assessing the chronic stress response and potential protective factors (i.e., family or social support) may assist in targeting appropriate interventions (22).

To address student concerns of reduced learning outcomes, the affected cohorts could be tracked in their future academic performance, NAVLE performance, and early graduate competency.

## Conclusions

The COVID-19 pandemic has brought many challenges and opportunities for growth in academia as well as other areas of life. Framing these experiences through the model of grief in response to uncertainty distress helps categorize reactions and possible interventions. Understanding the commonality of lived experiences can help individuals feel a sense of community while assisting colleges to best meet student needs. Providing clear communication, continued academic support, and normalizing mental healthcare as the pandemic and recovery continue will help mitigate the negative effects of these novel situations.

## Data Availability Statement

The datasets presented in this article are not readily available because of participant privacy concerns. Requests to access the datasets should be directed to Kimberly Carney, kimberly.carney@lmunet.edu.

## Ethics Statement

The studies involving human participants were reviewed and approved by Lincoln Memorial University Institutional Review Board. The patients/participants provided their written informed consent to participate in this study.

## Author Contributions

KC contributed to the conception and design of the study, organized the database with initial coding, performed statistical analysis, and wrote the first draft of the manuscript. RT and KC conducted the interviews and coding of data. RT wrote sections of the manuscript. Both authors contributed to manuscript revision, read, and approved the submitted version.

## Conflict of Interest

The authors declare that the research was conducted in the absence of any commercial or financial relationships that could be construed as a potential conflict of interest.
