# Supplementary material for: Grief in Response to Uncertainty Distress Among Veterinary Students During the Early Stages of the COVID-19 Pandemic
Source: Front Vet Sci. 2021 Jul 8;8:662198. doi: 10.3389/fvets.2021.662198 (PMC8295725; doi:10.3389/fvets.2021.662198)
Supplement: Supplementary file 1 [file Table_1.DOCX]

Grief and uncertainty distress among veterinary students during the early stages of the COVID-19 pandemic.

**Interview instrument**

(probes such as silence, nodding, “tell me more,” “how did you feel about that” will be added as needed to ensure depth and quality of response)

Thanks for meeting with me today. I really appreciate your time and yet another on-line commitment. Did you get a chance to read, sign, and return the consent form?

Any questions before we get started?

Which is your anticipated graduation year?

How has COVID 19 impacted your life?

Tell me five words that reflect your emotions about COVID--positive or negative.

How concerned are you about the COVID19 risk to you personally?

How has the social distancing/shelter in place affected you?

How strictly have you been following the recommendations?

Why?

What is the impact on your academic/professional life?

Social life? Family life?

What aspects of being on campus do you miss?

What have you learned you can easily live without?

Tell me about the change in stressors throughout the semester (BC-before corona and CE-corona era)

Stress management techniques you are using?

How do you feel about online classes or meetings?

Challenges?

Benefits?

What about online exams/assessments?

Do you feel like you have more free time? How are you using it or staying motivated?

You’ve shared a bit about _________ and ____________. Are there any other things you’d like to share?
